# Atypical, Clinically Silent, Locally Advanced Pheochromocytoma Revealing Von Hippel-Lindau Type 2C Phenotype: A Case Report

**DOI:** 10.7759/cureus.104523

**Published:** 2026-03-02

**Authors:** Zineb Eddebbarh, Zineb Serhane, Zineb El Azime, Mohammed Amine Essafi, Hayat Aynaou, Houda Salhi

**Affiliations:** 1 Department of Endocrinology, Diabetology, Metabolic Diseases, and Nutrition, Hassan II University Hospital, Fez, MAR

**Keywords:** chromogranin a, metastatic, neuroendocrine tumor, pheochromocytoma, von hippel-lindau disease

## Abstract

Pheochromocytomas are rare neuroendocrine tumors arising from chromaffin cells of the adrenal medulla. Malignancy is defined by the presence of distant metastases. Approximately a substantial proportion are associated with germline mutations, particularly in the context of Von Hippel-Lindau (VHL) syndrome. We report the case of a 60-year-old man diagnosed with a clinically silent locally advanced pheochromocytoma following a computed tomography-guided biopsy of a left retroperitoneal mass discovered during evaluation for persistent lower back pain.

Biochemical evaluation revealed normal 24-hour urinary metanephrines despite markedly elevated chromogranin A levels. Imaging demonstrated an 80 × 76 mm left adrenal mass with locoregional invasion. Histopathological and immunohistochemical analyses confirmed pheochromocytoma. ^123^I-metaiodobenzylguanidine scintigraphy showed increased uptake in the adrenal region. Genetic testing identified a pathogenic VHL mutation: NM_000551.4(VHL):c.508G>A, consistent with VHL type 2C phenotype. Systematic screening for other VHL-associated lesions was negative. This case highlights the diagnostic challenge posed by clinically silent pheochromocytomas and underscores the importance of genetic evaluation in atypical adrenal tumors.

## Introduction

Pheochromocytoma is a rare neuroendocrine tumor arising from chromaffin cells of the adrenal medulla and is typically characterized by catecholamine secretion [1]. Approximately 40% of cases are associated with germline mutations and may occur in the context of hereditary syndromes such as Von Hippel-Lindau (VHL) disease.

VHL syndrome is an autosomal dominant disorder predisposing to multiple tumors, including pheochromocytoma, which occurs in 10%-30% of patients depending on the subtype [2]. In VHL type 2C, pheochromocytoma may represent the sole clinical manifestation in the absence of other classical lesions such as retinal angiomas, central nervous system hemangioblastomas, or renal cell carcinoma [3].

Clinically silent pheochromocytomas, defined by the absence of typical catecholamine-related symptoms and sometimes normal biochemical findings, represent a diagnostic challenge and may delay appropriate management [4].

We report a case of a clinically silent locally advanced pheochromocytoma revealing VHL type 2C, highlighting the importance of genetic evaluation and careful diagnostic assessment in atypical adrenal tumors.

## Case presentation

A 60-year-old man with a four-year history of type 2 diabetes mellitus (treated with gliclazide 90 mg daily and metformin 2 g/day), hypertension controlled with calcium channel blockers, and chronic tobacco use presented with persistent lower back pain evolving over a 10-month period. He denied episodes of paroxysmal hypertension, palpitations, headaches, diaphoresis, or weight loss.

A thoracoabdominal-pelvic computed tomography (CT) scan performed to investigate chronic back pain revealed a left adrenal mass measuring 80 × 76 mm with evidence of locoregional extension into adjacent retroperitoneal structures. No distant lesions suggestive of metastases were identified. The patient was admitted for further evaluation.

On physical examination, the patient was normotensive, well hydrated, and without orthostatic hypotension. Abdominal examination revealed tenderness in the left flank without a palpable mass. No clinical signs suggestive of catecholamine excess were observed. Laboratory findings are summarized in Table 1.

**Table 1:** Biochemical and hormonal profile of the patient Plasma-free metanephrines were not available at our institution. Despite markedly elevated chromogranin A levels, 24-hour urinary metanephrines were within normal limits, and the patient had no symptoms suggestive of catecholamine excess. These findings supported the classification of the tumor as clinically silent DHEA-S: dehydroepiandrosterone sulfate

Test	Result	Reference range	Unit	Comment
24-hour urinary metanephrines	Within normal range	<0.9	mg/24 hours	No biochemical evidence of catecholamine hypersecretion
Chromogranin A	627	0-95	ng/mL	Elevated → consistent with neuroendocrine tumor
Morning cortisol	12	5-25	µg/dL	Normal
DHEA-S	Within normal range	35-430	µg/dL	Normal
Estradiol	Within normal range	<39	pg/mL	Normal
17-hydroxyprogesterone	Within normal range	0.2-1.3	ng/mL	Normal

Radiological findings

**Figure 1:**
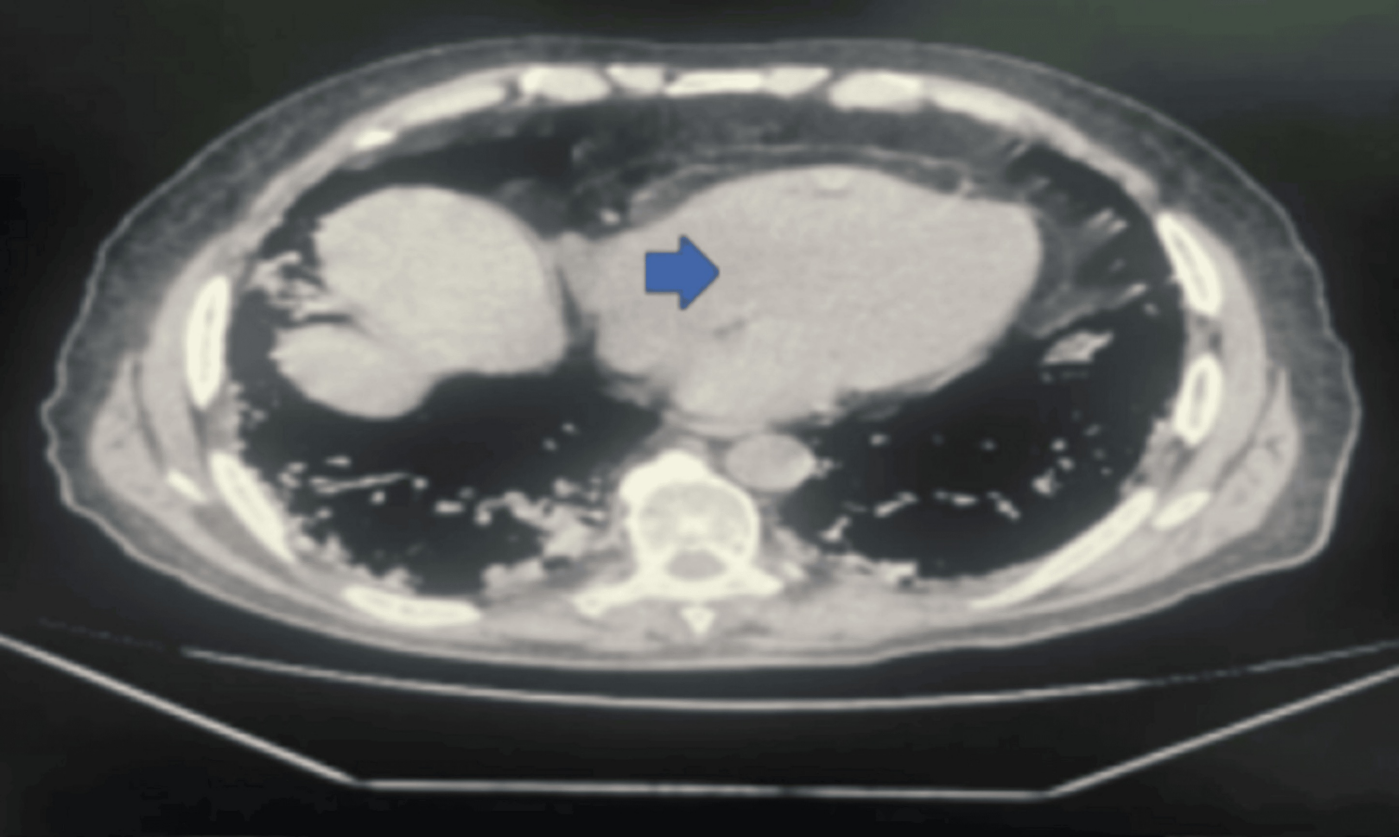
Axial CT scan showing a left paramedian retroperitoneal mass measuring 80 × 76 mm with locoregional invasion CT: computed tomography

A thoracoabdominal pelvic CT scan demonstrated a left paramedian retroperitoneal mass measuring 80 × 76 mm with locoregional invasion (Figure 1).

Histological diagnosis and biopsy considerations

At the time of biopsy, pheochromocytoma was not strongly suspected due to normal urinary metanephrines and absence of typical clinical manifestations. Consequently, no preprocedural alpha-adrenergic blockade was administered.

A CT-guided percutaneous biopsy of the retroperitoneal mass was performed without immediate hemodynamic complications. In retrospect, percutaneous biopsy of a suspected pheochromocytoma is generally contraindicated without adequate biochemical exclusion or alpha-blockade, and this approach may be considered exceptional.

**Figure 2:**
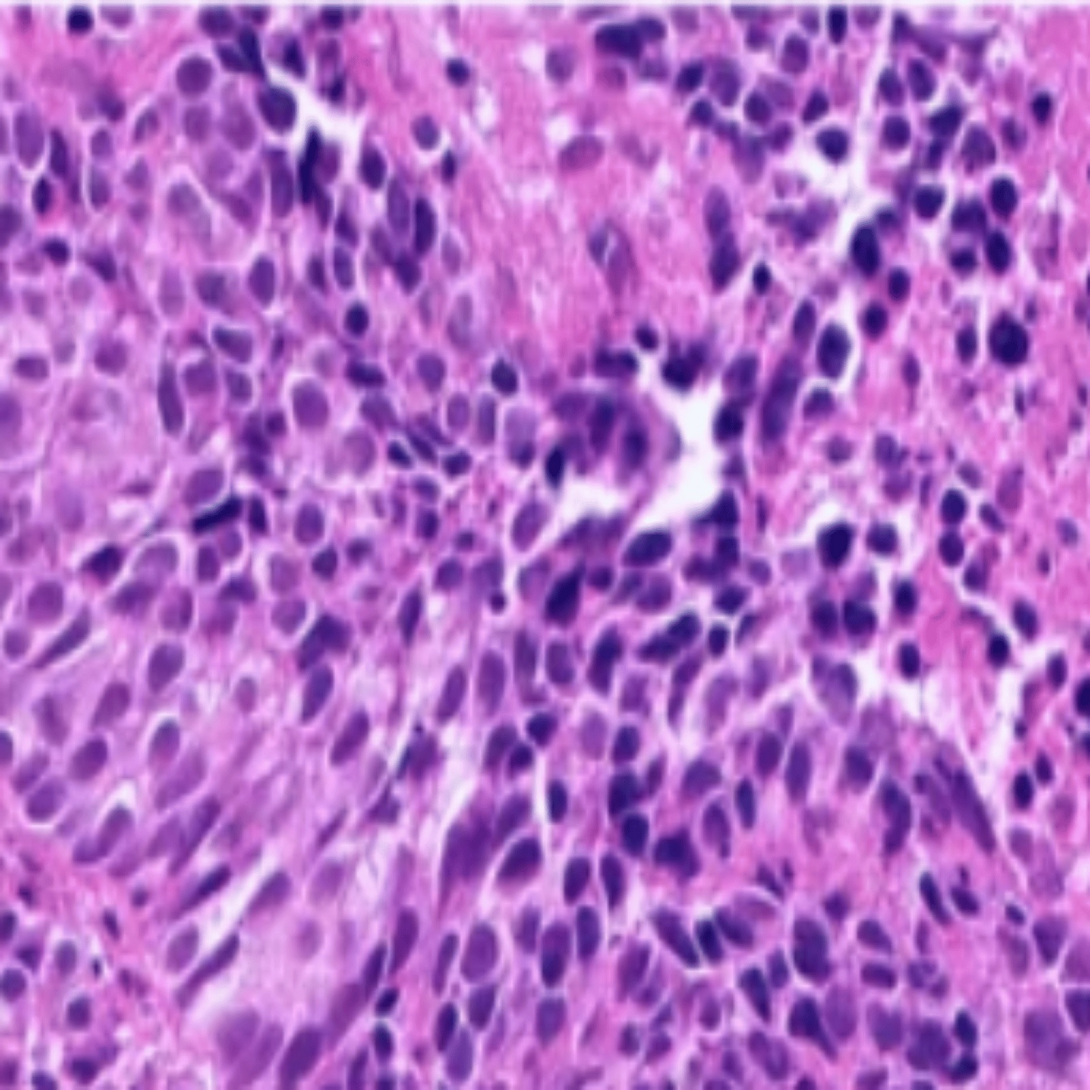
Immunohistochemical staining showing strong cytoplasmic positivity for chromogranin A in tumor cells (chromogranin A stain, original magnification ×400)

Histopathological examination demonstrated spindle-cell proliferation consistent with a fusocellular variant of pheochromocytoma. Immunohistochemical analysis showed strong cytoplasmic positivity for chromogranin A and synaptophysin, confirming the neuroendocrine origin of the tumor. Epithelial membrane antigen expression and the absence of cytokeratin staining excluded neuroendocrine carcinoma (Figure 2).

Functional imaging and genetic evaluation

**Figure 3:**
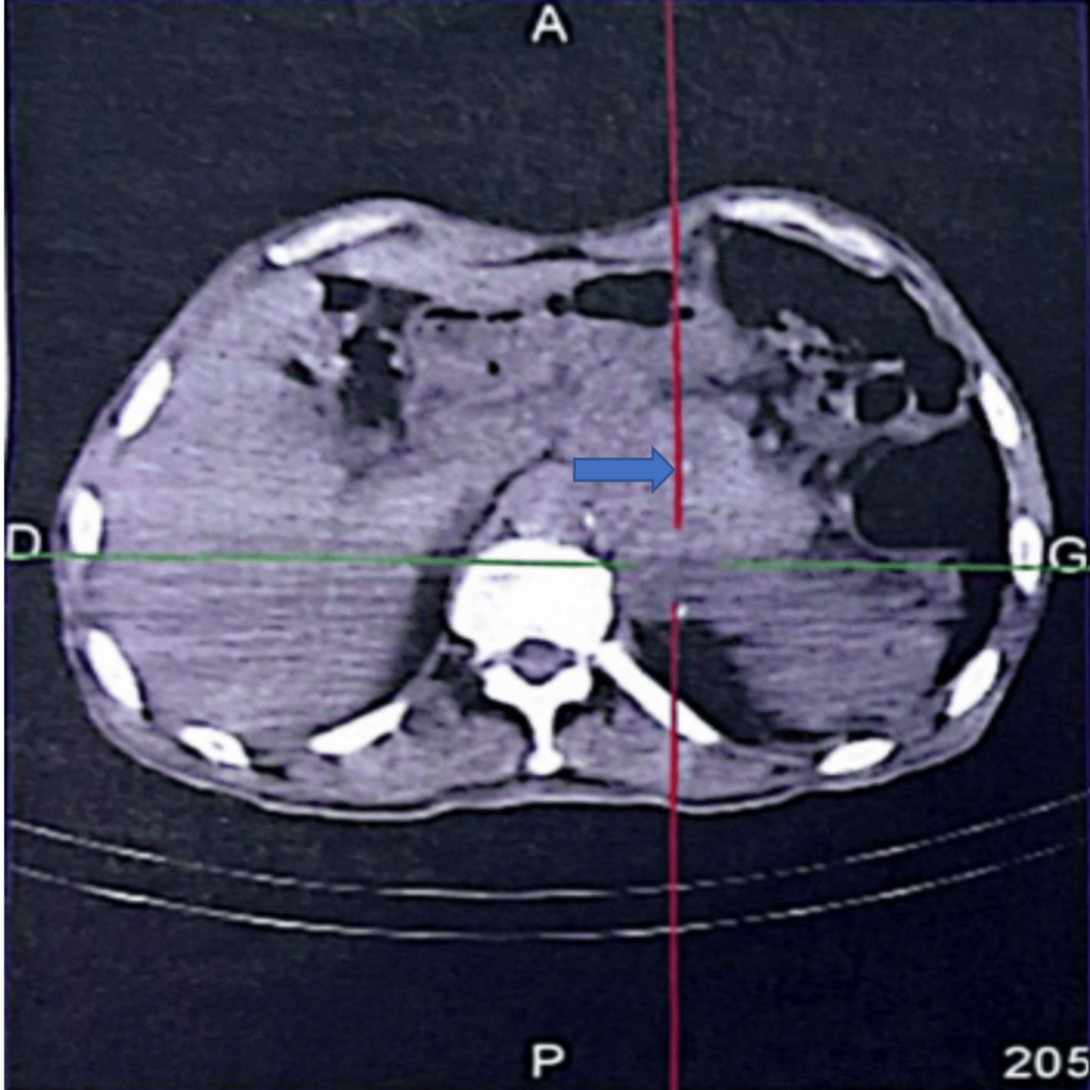
¹²³I-MIBG scintigraphy showing focal radiotracer uptake in the left adrenal region, consistent with pheochromocytoma ¹²³I-MIBG: iodine-123 metaiodobenzylguanidine

^123^I-metaiodobenzylguanidine scintigraphy demonstrated increased radiotracer uptake in the left adrenal region, consistent with pheochromocytoma. No distant metastatic lesions were detected (Figure 3).

Genetic testing revealed a pathogenic missense mutation in the VHL gene: NM_000551.4(VHL):c.508G>A, consistent with a VHL type 2C phenotype. Comprehensive screening for other VHL-associated manifestations, including retinal angiomas, central nervous system hemangioblastomas, and renal cell carcinoma, was negative.

Management and outcome

Given the tumor size and significant locoregional invasion, surgical resection was deemed unfeasible after multidisciplinary evaluation. The patient received locoregional management and oncologic follow-up.

During disease progression, he developed cholestatic jaundice secondary to tumor invasion of the distal bile duct and, unfortunately, died, reflecting the aggressive nature of locally advanced pheochromocytoma despite the absence of distant metastases.

## Discussion

Pheochromocytomas are rare catecholamine-producing neuroendocrine tumors arising from chromaffin cells of the adrenal medulla, with an estimated annual incidence of two to eight cases per million person-years. Although classically associated with paroxysmal hypertension, headaches, palpitations, and diaphoresis, clinical presentation can be highly variable [1,2]. In some cases, tumors may lack overt symptoms of catecholamine excess, leading to delayed recognition or incidental discovery.

Clinically silent pheochromocytomas represent a diagnostic challenge. These tumors may present without typical adrenergic manifestations and, in rare instances, may even demonstrate normal biochemical screening results [3]. Current diagnostic guidelines recommend measurement of plasma-free metanephrines or 24-hour urinary fractionated metanephrines as first-line tests [4]. In our patient, 24-hour urinary metanephrines were within normal limits, and classic symptoms were absent, supporting the classification as a clinically silent pheochromocytoma. The markedly elevated chromogranin A level reflected neuroendocrine tumor burden rather than active catecholamine hypersecretion.

Tumor size is an important prognostic consideration. Lesions larger than 5-6 cm are associated with a higher risk of aggressive behavior [5]. In this case, the adrenal mass measured 80 mm and demonstrated significant locoregional invasion. However, no distant metastases were identified on imaging studies. It is important to emphasize that malignancy in pheochromocytoma is defined exclusively by the presence of distant metastases rather than by local invasion or histological features alone [6]. Therefore, this tumor should be considered locally advanced rather than metastatic [7].

Approximately 40% of pheochromocytomas are associated with germline mutations. VHL syndrome is one of the most frequently implicated hereditary conditions [8]. VHL type 2C is a rare subtype characterized predominantly by pheochromocytoma in the absence of other classical VHL-associated lesions such as retinal angiomas, central nervous system hemangioblastomas, or renal cell carcinoma [9]. In our patient, identification of the pathogenic mutation NM_000551.4(VHL):c.508G>A confirmed the diagnosis of VHL and justified systematic screening for additional manifestations, which remained negative. This presentation is consistent with a VHL type 2C phenotype.

The decision to perform a percutaneous biopsy in adrenal masses must be approached with caution. Biopsy of a suspected pheochromocytoma is generally contraindicated without adequate biochemical exclusion or preprocedural alpha-adrenergic blockade due to the risk of catecholamine crisis [10]. In our case, the absence of clinical suspicion and normal urinary metanephrines led to biopsy without prior alpha-blockade, and no immediate complications occurred. Nevertheless, this approach should be considered exceptional.

Management of pheochromocytoma depends on tumor stage and resectability. Surgical resection remains the standard of care for localized tumors [11]. However, in cases of locally advanced disease with significant invasion of surrounding structures, complete resection may not be feasible, necessitating a multidisciplinary approach. Long-term surveillance is essential, particularly in hereditary cases such as VHL, due to the risk of recurrence or development of additional tumors.

This case illustrates the diagnostic complexity of clinically silent pheochromocytoma and highlights the importance of considering hereditary syndromes in atypical adrenal masses. It also underscores the limitations of relying solely on biochemical testing and reinforces the value of histopathological confirmation and genetic analysis in selected cases.

## Conclusions

This case highlights the diagnostic challenges posed by clinically silent pheochromocytomas, particularly when biochemical screening results are within normal limits. The absence of classic catecholamine-related symptoms may delay recognition and complicate management decisions.

The identification of the pathogenic VHL mutation NM_000551.4(VHL):c.508G>A confirmed an underlying VHL type 2C phenotype, in which pheochromocytoma may represent the sole clinical manifestation. This underscores the importance of systematic genetic evaluation in patients presenting with atypical or large adrenal tumors.

Our case also emphasizes the need for careful consideration before performing percutaneous biopsy of adrenal masses, especially when pheochromocytoma has not been definitively excluded. Early multidisciplinary assessment and appropriate genetic counseling remain essential to optimize management and long-term surveillance in hereditary cases.
